# Explaining Adherence to American Academy of Pediatrics Screen Time Recommendations With Caregiver Awareness and Parental Motivation Factors: Mixed Methods Study

**DOI:** 10.2196/29102

**Published:** 2022-04-05

**Authors:** Shea M Lammers, Rebecca J Woods, Sean E Brotherson, James E Deal, Carrie Anne Platt

**Affiliations:** 1 Research Institute Children's Minnesota Minneapolis, MN United States; 2 Casimir St. Cloud, MN United States; 3 Department of Human Development and Family Science North Dakota State University Fargo, ND United States; 4 Department of Communication North Dakota State University Fargo, ND United States

**Keywords:** infancy, screen time, screen time recommendations, mothers and infants, American Academy of Pediatrics recommendations, parental motivations

## Abstract

**Background:**

With the increasing integration of technology into society, it is advisable that researchers explore the effects of repeated digital media exposure on our most vulnerable population—infants. Excessive screen time during infancy has been linked to delays in language, literacy, and self-regulation.

**Objective:**

This study explores the awareness of and adherence to the American Academy of Pediatrics’ (AAP) recommendations related to avoiding screen time for infants younger than 2 years and the motivational factors associated with screen time exposure.

**Methods:**

A mixed methods survey design was used to gather responses from 178 mothers of infants younger than 2 years. The measures included infant screen time use and duration, maternal awareness of screen time use recommendations, and motivations related to screen time exposure. A variety of statistical procedures were used to explore associations between caregiver awareness of and adherence to AAP guidelines for screen time exposure, motivations related to screen time for infants, and the duration of infant screen time exposure.

**Results:**

The results indicated that 62.2% (111/178) of mothers were aware of the AAP screen time recommendations, but only 46.1% (82/178) could cite them accurately, and most mothers learned of them via the internet or from a medical professional. Mothers who were aware of the guidelines allowed significantly less screen time for infants than those who were unaware (*P*=.03). In addition, parents who adhered to the AAP guidelines reported significantly less infant screen time per day than those who did not adhere (*P*<.001). Among mothers who reported not adhering to the guidelines, the greatest motivation for allowing screen time was perceived educational benefits. Less educated mothers rated an infant’s relaxation as a motivational factor in allowing screen time significantly higher than more highly educated mothers (*P*=.048). The regression analysis indicated that none of the parental motivation factors predicted daily infant screen time.

**Conclusions:**

These results indicate 2 key approaches to improving adherence to screen time recommendations. First, the awareness of the AAP recommendations needs to be increased, which tends to improve adherence. Second, the myth that screen time can be educational for infants needs to be dispelled.

## Introduction

### Background

Exposure to screen time during infancy has become prevalent in the past few decades as advances in technology have merged with educational and entertainment products targeting infants and their caregivers. Informed by research showing that screen time can be detrimental to infant development, researchers and pediatricians recommend that children younger than 2 years be strictly limited in their screen time exposure or even better, have no sedentary exposure to electronic media at all [1-6]. Recommendations by entities such as the American Academy of Pediatrics (AAP) are intended to provide useful guidelines for parents and caregivers when making decisions about how to manage screen time exposure for young children [5]. However, their utility is limited if caregivers are not aware of or do not adhere to the guidelines. To address this problem, we explored mothers’ awareness of and adherence to the AAP’s recommendations and the motivational factors associated with screen time exposure.

### Problem Statement: Adverse Effects of Screen Time Exposure for Infants

Previous research has demonstrated that infants and toddlers gain more developmentally beneficial skills through play time with physical objects than through devices that use screens [2-4,6-11]. Screen time deprives infants from learning and developing adaptive skills that can only be obtained through human interaction, and it does not allow them the creative freedom experienced during free play [5,12]. A rapidly growing body of literature has linked screen time to delays in both language development and emotional regulation [3,4,9,12-15]. Even 1 hour of television viewing can negatively affect an infant’s language capacity, as an estimated 52 minutes of interaction between the infant and their caregiver are lost during that 1 hour [12]. The presence of background media has also been shown to reduce caregiver interactions with infants [16,17].

Empirical research on educational media suggests limited benefits for language learning, prompting some researchers to conclude that there are no beneficial effects of watching programs for children younger than 2 years [1,4,10,18]. Although many parents believe that educational media are helpful for language learning, research suggests that infants are not able to learn from screen time the way they learn from real-life experiences. Vandewater [19] found no differences in language development between infants (aged 8-15 months) who were regularly shown an infant-directed language DVD and those who were not. In a study of 6- to 36-month-old children, Taylor et al [20] found that reading was associated with a larger vocabulary, whereas screen media had no impact on vocabulary. The one type of media that is seen as making a positive contribution to development is live human interaction via video chat, which the AAP classifies as an exception to the *no screen time* rule [13]. The reason video chat is an exception is that a substantial amount of contingent interaction with the infant in the form of communication takes place during the call [13].

When screen time reduces interaction with caregivers and other children, deficits in self-regulation and other forms of socioemotional learning can result. Self-regulation is a preacademic skill that undergoes great gains during infancy and toddlerhood [21]. When self-regulation is poorly developed, an individual will struggle to stay focused on a task, lack the ability to inhibit automatic responses, and have a decreased capacity for long-term and working memory [21]. Screen time supplants the human interaction necessary to develop these fundamental skills [4,12,22]. Both experimental and large-scale longitudinal survey studies have found that screen time negatively impacts self-regulation abilities [22-24]. Although screen time may be moderately engaging for young children, the effects of screen time are likely to be detrimental across developmental domains.

### Caregiver Awareness and Motivations as Impediments to the Current Solution

To minimize the adverse impact of screen time on infants, multiple professional organizations recommend avoiding infant screen time exposure as much as possible. However, this approach has not resulted in widespread reductions in infant screen time. Despite research documenting the negative effects of screen time and the AAP recommending minimal screen media use for children younger than 2 years, parents continue to allow and even encourage its use by their infants and toddlers. Currently, it is unclear whether the lack of adherence stems from caregivers being unaware of these guidelines or believing that screen time has benefits for their families. To support more optimal child outcomes, our study explores the impact of awareness and caregiver motivation for screen time on infants’ screen time durations. By better understanding the context in which caregivers receive screen time guidelines, medical organizations and practitioners will have a better sense of how to advocate for reduced screen time more effectively and increase adherence to recommendations.

Caregivers use a variety of sources to gather information regarding the process of raising children, including for both immediate problems and general advice [25-27]. Through a survey of 1240 parents, Radey and Randolph [28] found that parents typically gather information from multiple sources when looking for general parenting knowledge, including a combination of professional, nonprofessional, and media sources. Looking at the relative impact of various sources through an interview-based study, van der Gugten et al [27] found that although parents used the internet most frequently to gather information about a child’s issues, they relied on physician recommendations to alleviate worries.

Although these few studies explore sources that caregivers commonly use, studies on parental awareness of the AAP screen time recommendations have produced mixed results. Funk et al [29] surveyed 94 parents of preschool-aged children and found that just one-third (34%) of them were able to correctly identify current screen media recommendations for young children. In contrast, Adamiak [30] found that 76% of a sample of 126 parents of preschool-aged children were aware of age-specific AAP recommendations for media use. Our study adds to this scholarship by testing relationships between awareness of and adherence to guidelines.

A lack of awareness may not be the only factor associated with higher infant exposure to screen time. Parents exhibit a variety of motives in exposing their children to screen time in the early years, restricting access sometimes, whereas encouraging such use at other times. [31]. Cingel and Krcmar [32] have called for more work in this area, noting that “little work has systematically examined parents’ motives for media use for their young children.” In their study, parents ranked child enjoyment of media, perceived educational benefits, and need to do other tasks as the most important motives for facilitating child media use [32]. Brown and Smolenaers [2] used interviews to investigate the motivational factors behind exposing children younger than 2 years to screen time, finding that child enjoyment, availability of screens, need to do other tasks, and coping with child upset were all seen as reasons for permitting infant screen time use. Our study investigates motivational factors across a larger sample, testing for relationships between motivational factors and caregivers’ adherence to screen time recommendations.

### Study Context and Research Questions

Major professional organizations focused on child health and well-being, specifically the AAP, have conducted reviews of research and published guidelines intended to foster best practices in raising young children [29,33]. However, the implementation of such recommended practices is contingent on parental awareness of, adherence to, and motivations related to such guidelines. Therefore, in this study, our goal is to recruit a sample of parents with infants younger than 2 years and assess these variables in relation to the topic of screen media recommendations laid out by the AAP.

Given the nascent stage of research on parental awareness and motivation for infant screen time use, we seek to apply a mixed methods survey approach (quantitative and qualitative elements) to further explore the variables of awareness, adherence, and motivation for screen media exposure among parents (mothers) of infants (age ≤2 years). We seek to investigate several research questions (RQs) as follows:

- RQ 1: What is the level of awareness that parent caregivers express related to AAP’s recommendations on limiting screen time exposure for infants?
- RQ 2: What is the level of adherence to AAP screen time recommendations by parent caregivers and does the level of adherence influence infant screen time exposure?
- RQ 3: What is the association, if any, between parent caregivers’ awareness of AAP screen time recommendations for infants and adherence to such guidelines in parental behavior?
- RQ 4: In circumstances of nonadherence to AAP screen time recommendations for infants, what are mothers’ motivations for allowing their young children to use screen media and does maternal education influence such motives?
- RQ 5: Do parental motivation factors among parents not adhering to AAP screen time recommendations predict screen time exposure for their infants?

In exploring these questions, we anticipate some possibilities based on previous research and pragmatic considerations. With regard to awareness of AAP screen time recommendations, we suspect that although many caregivers have likely heard about such recommendations, there might be an inaccurate understanding of the guidelines [2]. We use a qualitative approach to further assess this possibility. Furthermore, we feel it is likely that some mothers would allow or facilitate screen time (ie, not adhere to AAP recommendations) and thus want to better understand how awareness is linked with adherence. Finally, as previous research has articulated some parental motivations for facilitating screen time exposure for young children [2,32], we seek to understand how such motives may take precedence over a desire to follow AAP screen time recommendations.

## Methods

### Design

Information for this study was gathered using a descriptive, cross-sectional design with a mixed measures approach (questionnaire) among a population in the upper midwestern United States. The survey included both quantitative and qualitative elements and was distributed via a web-based platform (Qualtrics; Qualtrics International Inc) to parents of at least one infant child between the ages of 0 and 2 years. To maintain consistency with previous research discussed in the literature review, fathers were excluded from our study, as most of the work in this area is only examined with primary caregivers, who are usually mothers. Collaborating entities in the research project were the Infant Cognitive Development Lab at North Dakota State University (NDSU), the NDSU Extension Service, and the Early Head Start program of North Dakota.

### Ethics Approval

Approval for the study was obtained from the institutional review board of NDSU (HE19122).

### Participants

A total of 178 mothers of an infant younger than 2 years were selected for inclusion in the final sample for the study. Potential participants were informed of the study and recruited for involvement via information shared through one of the collaborating entities. To be eligible for inclusion in the study, participants needed to be a female primary caregiver and care for an infant aged 0-23 months. Initially, a total of 220 individuals completed the survey. Individuals excluded from the final sample were those who were male (34/220, 15.5%), those who did not categorize themselves as primary caregivers (3/220, 1.4%), and those who did not complete the survey questions beyond the demographics section (5/220, 2.3%). Once these individuals were excluded, 80.9% (178/220) of the female caregivers of infants aged 0-2 years remained in the final sample.

Participants responded to a variety of demographic questions that included age, relationship to the infant, age of the infant, number of children, race or ethnicity, partnership status, education level, employment status, and annual family income. Of the 178 primary caregivers, nearly all reported their relationship to the target infant as biological mother or adoptive mother (174/178, 97.7%), whereas 1 (0.6%) each reported as stepmother, grandmother, aunt, and foster parent. Caregivers’ ages ranged from 18 to 56 years (mean 29.5, SD 5.57 years). Reported mean age of the focal infant was 12.5 (SD 6.62) months. In addition, participants had an average of 2.07 (SD 1.07) other children in the home. Remaining participant characteristics are presented in Table 1. It is important to note that participants were allowed to opt out of the questions given in the survey, including annual family income.

**Table 1:** Characteristics of caregivers of infants aged 0-2 years (N=178).

Caregiver characteristic		Value, n (%)
**Race or ethnicity**		
	White	153 (85.9)
	Black or African American	6 (3.4)
	Native American or native Alaskan	8 (4.5)
	Asian	4 (2.2)
	Hispanic or Latino	4 (2.2)
	Multiracial	3 (1.7)
**Partnership status**		
	Married	115 (64.6)
	Single	24 (13.5)
	Significant other (not engaged)	21 (11.8)
	Significant other (engaged)	14 (7.9)
	Divorced or separated	4 (2.2)
**Education level**		
	High school or equivalent degree or less	33 (18.5)
	Some college or associate degree	52 (29.2)
	Bachelor’s degree	58 (32.6)
	Postgraduate degree	32 (17.9)
	Other	3 (1.7)
**Employment status**		
	Not seeking outside employment	39 (21.9)
	Seeking employment	6 (3.4)
	Employed <25 hours per week	18 (10.1)
	Employed 26-39 hours per week	22 (12.4)
	Employed >40 hours per week	93 (52.2)
**Annual family income (US $; n=173^a^)**		
	0-20,000	34 (19.6)
	20,001-40,000	29 (16.7)
	40,001-60,000	27 (15.6)
	60,001-80,000	24 (13.9)
	80,001-100,000	29 (16.8)
	>100,001	30 (17.3)

^a^Five mothers opted to not provide their income details.

### Procedure

Collaborating partners distributed study information by emailing a project link and QR code to contact families, displaying flyers around the local area or offices, supporting local recruitment events, and making the survey available for eligible participants on an accessible computer in their office locations. A convenience sampling strategy was used and supplemented by purposive sampling with families eligible for the Early Head Start program to reach a population with broader socioeconomic backgrounds. Data were collected from May 2019 to January 2020.

The survey was made available electronically via Qualtrics, and participants were able to reach it via an email link or QR code specific to the survey. Mothers took the survey in a location of their choice, including the Infant Cognitive Development Lab or the Early Head Start program offices. Participants were prompted with a brief paragraph explaining the purpose of the study, an informed consent page, and a questionnaire link. The survey took approximately 15-25 minutes for participants to complete. Upon completion, participants were thanked and then provided a code word, which they could use to redeem for a compensation baby item at any of the collaboration sites.

### Measures

#### Overview

Participant information was gathered through completion of a questionnaire that included questions regarding participant characteristics, infant screen media exposure, parental knowledge of media guidelines, and parental motivation related to infant media use. Responses were collected in various formats including Likert-type scales, short entry or drop-down lists, and short essay responses. This combination of approaches allowed mothers to answer some of the questions in their own words and provide insight into their awareness and thought patterns. A number of specific measures were used to assess participants’ responses.

#### Infant Screen Time Exposure

To measure screen time exposure of infants, participants were asked to report the duration of the focal infant’s average daily screen time use in multiple-choice format with 5 options from 1 (*0-1 hour*) to 5 (*>4 hours*). A total of two questions were asked (4 in total) for screen time duration, 1 related to television and 1 related to other digital devices, for both the average *weekday* and the average *weekend day*. An example question was as follows: “On an average *weekday*, how much time does your child spend on a digital device (e.g., cellphone or tablet) while under your supervision?” Screen time was measured in 1-hour increments (eg, *0-1 hour*) with no true zero. As the responses were recorded in ranges of time, results should be interpreted as a median approximation of time with every 0.5 equating to 30 minutes of screen time. To calculate an infant’s average screen time per day, we computed a variable by adding the reported estimate of screen time on each of 2 different types of devices (eg, television) that infants were exposed to on an average weekday and multiplying this value by 5. Then, the estimated amount of screen time per weekend day was multiplied by 2. Next, these 2 values were added together and divided by 7 to give an overall daily average (with a possible range from 0-10 hours). Mothers who selected *0-1 hour* and also responded “I never allow screen time” were coded as 0 hours. This computed *screen time per day* variable was used as a dependent variable for multiple analyses. The questions on infant screen time exposure were developed specifically for this project.

#### Caregiver Awareness of Infant Screen Time Use Recommendations

To assess caregiver awareness of the current AAP recommendations on screen time for children aged <2 years, participants were asked a multiple-choice question: “How did you find out about the American Academy of Pediatrics’ recommendation?” Six possible options included lack of awareness, unlisted source, or four other possibilities (medical professional, other community member, web, and book). First, responses were coded dichotomously as *aware* or *unaware* based on their self-report, with all participants indicating “I did not know about the recommendation” being coded as *unaware*, whereas the remaining responses (eg, “I read it online” and “A medical professional informed me”) being coded as *aware*. Next, further analysis was conducted, and responses were coded into 6 categories reflecting the accuracy of awareness and confidence in their assessment of the guidelines by examining open-ended responses. Participants were asked an additional open-ended question: “In your own words, what is the current American Academy of Pediatrics’ recommendation for use of digital media or television by children under the age of two?” This question allowed caregivers to reveal their knowledge about the current guidelines. The questions on caregiver awareness of AAP recommendations were developed specifically for this project.

#### Caregiver Adherence to Infant Screen Time Use Recommendations

To assess caregiver adherence to AAP infant screen time recommendations, participants were given a clear statement of current AAP guidelines on the topic and informed that parents often vary in following this guideline. Then, caregivers were asked, “How often do you adhere to this recommendation?” Response options ranged on a 5-point scale from 1 (*never*) to 5 (*always*). On the basis of their responses, skip logic led to the next prompt that asked participants to explain if they adhere, partly adhere, or do not adhere to the AAP recommendations in their own words. For analysis, adherence to the AAP’s guideline was recoded into a dichotomous variable as *adherent* or *nonadherent*. Caregivers who followed the AAP guideline *most of the time* (rating=4) or *always* (rating=5) were coded as *adhering*, but those who reported adhering *never* (rating=1), *sometimes* (rating=2), or *about half the time* (rating=3) were coded as *not adhering*. The questions on caregiver adherence to AAP recommendations were also developed specifically for this project.

#### Parental Motivations Scale

A slightly adapted version of the Parental Motivations Scale [32] was used in this study. The Parental Motivations Scale by Cingel and Krcmar [32] was developed based on a qualitative interview study on parent motivations in relation to screen time for young children conducted by Rideout et al [34]. The Parental Motivations Scale was originally validated through an exploratory factor analysis that used a varimax rotation, which identified 5 dimensions with eigenvalues >1 and Cronbach α scores ranging from .77 to .92 [32]. These dimensions were *chores* (to get chores done), *education* (for educational purposes), *reward* (as a reward for good behavior), *relax* (to help children calm down or relax), and *enjoyment* (because the child enjoys or asks for screen time). The scale starts with the prompt “I let my child use media...” and then lists a variety of reasons, such as “...because it is educational” or “...to help alleviate my stress.” A higher score on each scale construct indicates that respondents are more likely to let children use media based on that particular parental motive. Participants responded using an expanded 19-item Likert scale matrix table rating each item from 1 (*strongly disagree*) to 7 (*strongly agree*). Owing to technology advancement and evolving parental standards, we solicited input from local parent groups to identify other potential motivators. On the basis of this input, we created 3 exploratory scale items. These items fit into the factors of chores and education (Multimedia Appendix 1). One additional question was added as a screening question, which allowed mothers to indicate that they never allow their infants screen time. Reliability analysis for this sample for each factor indicated high reliability (Cronbach α=.82-.93). When the exploratory questions were included, internal consistency for the education factor remained the same (Cronbach α=.92), but that for the chores factor increased from the original Cronbach α=.88 to Cronbach α=.92. Thus, the additional questions improved the internal consistency of the chores subscale.

### Analysis

Results were calculated using the final sample of female caregivers (N=178). However, we noted that beyond the screening questions (eg, consent, having an infant younger than 2 years, gender of the participant, and indicating they were the primary caregiver), questions were elective, meaning that some caregivers may have opted out of answering certain questions. All analyses used raw scores and unedited short-answer responses. All quantitative analyses were conducted using SPSS 27.

For RQ 1, descriptive statistics were used to identify the number of caregivers who were aware of the AAP’s recommendations. Furthermore, brief thematic analyses of short-answer responses were coded on the basis of correctness and confidence level. Coding was completed by a primary investigator (SML) manually inserting responses into the corresponding categories within a Microsoft Excel file. After a thorough review of the coding was conducted by the secondary investigator (CAP), percentages were calculated for each category of correctness and confidence level. Finally, caregivers identified the sources from which they learned the AAP’s recommendations, and descriptive statistics provided the frequencies of each source.

For RQ 2, caregivers were told the current screen time guidelines for infants aged <2 years and asked about their adherence practices. Descriptive statistics were used to analyze the caregivers’ adherence practices. Responses were dichotomized in which ratings of 4 (*most of the time*) or 5 (*always*) were coded as *adhering*, whereas ratings from 1 (*never*) to 3 (*about half the time*) were coded as *not adhering*. Next, a univariate ANOVA was used to compare adherent and nonadherent mothers’ infant screen times.

Next, for RQ 3, we sought to identify whether there was an association between awareness of the AAP’s recommendations and parental adherence. Each of the relevant variables was coded as a dichotomous variable in this analysis, and a chi-square analysis was used to explore whether difference between adherent and nonadherent caregivers was significant owing to awareness. Furthermore, a univariate ANOVA was used to examine potential contrasts in infant screen time exposure between caregivers who were aware and those who were unaware of the guidelines.

For RQ 4, when examining parental motivation factors related to infant screen time exposure, a filter was applied in which only caregivers who indicated infant exposure to screen time were analyzed (86/172, 50%). Descriptive statistics were used to investigate the participants’ ratings for each parental motivation factor. Maternal education was another variable explored in this section, and this item was recoded dichotomously for analysis purposes (eg, *low education level* was defined as lesser than a bachelor’s degree and *high education level* was defined as a bachelor’s degree or more). Independent sample *t* tests (2-tailed) were used to identify any differences among parental motivation factors based on caregiver’s education level.

Finally, for RQ 5, we sought to identify the parental motivation predictors of infant screen time exposure. Linear regressions were conducted on each parental motivation factor with respect to infant screen time exposure as a dependent variable.

## Results

### Demographics

Mothers completed a number of questions in the survey that collected information about participant characteristics. Specific items included age, relationship to the infant, age of the infant, number of children, race or ethnicity, partnership status, education level, employment status, and annual family income.

### RQ 1: Mothers’ Awareness of the AAP’s Recommendations on Infant Screen Time

Of the 178 participants, 172 (96.6%) participants responded to the survey question exploring their awareness of the current AAP’s recommendations on screen time for children younger than 2 years. Descriptive statistics indicated that many mothers were aware of the AAP’s screen time recommendations for infants (107/172, 62.2%). We conducted further qualitative analysis of responses to the question, “In your own words, what is the current American Academy of Pediatrics’ recommendation for use of digital media or television by children under the age of two?”

Of those who responded, 55.8% (96/172) showed full or partial awareness of the AAP screen time recommendations that was accurate. Results of this analysis demonstrate that 38.4% (66/172) of mothers knew the guidelines confidently and in their entirety (eg, “No screen time under the age of two” and “video chat with family members is ok, [but] should be limited. Everything [else] should be avoided.”). It is important to note that 7.6% (13/172) of mothers were correct but not confident in their knowledge of the guidelines (eg, “I believe it says very minimal or none?” and “I have no idea but I would guess none”). In addition, 9.9% (17/172) of mothers were partially correct (eg, “no TV at all” and “limit screen time or not have it at all”). The qualitative analysis further revealed that 30.2% (52/172) of mothers did not know the recommendations (eg, “I don’t know” and “less than one hour per day”). Finally, 14.5% (25/172) of mothers failed to answer the question, instead they either expressed their opinions or knowledge on the topic (16/25, 64%; eg, “Children learn best through play not media and videos” and “Unrealistic”) or gave nonapplicable responses (8/25, 32%; eg, “?” or “4-month-old baby”).

Participants responded to a follow-up question regarding how they learned of the AAP’s guidelines on infant screen time exposure. A substantial portion of mothers indicated they did not know about the recommendations at all (65/172, 37.8%); however, 3 out of 5 caregivers noted that they learned about the guidelines from a variety of sources (107/172, 62.2%). Most mothers in this group read about the AAP recommendations on the web (41/172, 23.8%), closely followed by being informed by a medical professional (38/172, 22.1%), and then followed by awareness via other sources such as news, Facebook, or childcare centers and so on (20/172, 11.6%). A few respondents learned the guidelines from someone other than a medical professional (6/172, 3.5%) or they read about them in a book (2/172, 1.2%).

### RQ 2: Adherence to the AAP’s Recommendations on Infant Screen Time

A second RQ investigated the adherence of infant caregivers to AAP screen time recommendations and whether such adherence influences daily infant screen time exposure. Participants (172/178, 96.6%) read a statement that clearly stated the AAP recommendations (eg, “children under the age of two should not use any digital media or watch television”), a sentence that explained parents vary in adherence to this guideline, and then were asked how often they adhered to this recommendation on a 5-point scale ranging from 1 (*never*) to 5 (*always*).

Adherence to the AAP’s recommendations on screen time exposure for infants was recoded into a dichotomous variable as *adherent* or *nonadherent*. Mothers who responded that they followed the screen time guidelines with ratings of 4 (*most of the time*) or 5 (*always*) were coded as *adhering*, whereas mothers who indicated following the guideline with ratings from 1 (*never*) to 3 (*about half the time*) were coded as *not adhering*. Descriptive statistics revealed that mothers who adhered to the AAP’s recommendations on infant screen time were exactly comparable in numbers with mothers who did not adhere (both 86/172, 50%), whereas 3.5% (6/172) of the mothers declined to answer whether they adhered to the guidelines.

To investigate the effect of caregiver adherence to the AAP recommendations on self-reported screen time exposure for infants, a univariate ANOVA was conducted to compare adherent and nonadherent mothers. The univariate ANOVA yielded a significant difference in infants’ average daily screen time exposure between parents who adhere and those who do not adhere to the AAP’s guidelines (*F*_1,169_=22.55; *P*<.001; η_p_^2^=0.12). Mothers who reported adhering to the guidelines indicated lower amounts of infant screen time per day (mean 0.65, SD 0.48 hours/day) compared with mothers who reported not adhering to the guidelines (mean 1.25, SD 1.06 hours/day), suggesting both higher levels of screen time exposure and greater variance in such exposure for children in households not adhering to AAP guidelines on the topic.

### RQ 3: Association Between Caregiver Awareness, Caregiver Adherence, and Infant Screen Time

The next RQ in this study explored whether there is any association between caregiver awareness of AAP screen time recommendations for infants and adherence to such guidelines in parental behavior. To further examine this question, we conducted a chi-square analysis of the association between caregiver awareness of AAP screen time recommendations and adherence to such AAP guidelines in allowing infant screen time exposure.

Chi-square analysis indicated that there was a significant association between parents’ awareness of the AAP screen time guidelines and parents’ adherence to them (172/178, 96.6%; *χ*^2^_1_=10.9; *P*<.001; Cramer *V*=0.25). Of the caregivers who were aware of the AAP’s guidelines (107/172, 62.2%), 59.8% (64/107) of them indicated that they adhere to the recommendations. In contrast, of those who were unaware of the AAP’s recommendations (65/172, 37.8%), only 34% (22/65) of the mothers reported that they adhered to the guidelines. This finding on caregiver awareness was further supported by a univariate ANOVA with daily infant screen time as the dependent variable. Results indicated that mothers who were aware of the guidelines allowed significantly less screen time (mean 0.84, SD 0.90 hours/day) than mothers who were not aware of the guidelines (mean 1.13, SD 0.79 hours/day; *F*_1,169_=4.63; *P*=.03; η_p_^2^=0.03).

### RQ 4: Parental Motivation Factors Related to Screen Time Exposure for Infants and Maternal Education

The next RQ explored parental motivation factors for allowing their infant to use screen media in circumstances of nonadherence to the AAP screen time recommendations for young children. In addition, we investigated whether maternal education influences such motives.

Using the subsample of caregivers who reported not adhering to the AAP guidelines (86/172, 50%), descriptive statistics were computed for each of the 5 parental motivation factors developed by Cingel and Krcmar [32] in their Parental Motivations Scale (scale of 1-7). A higher score on a specific motivation factor indicated a greater likelihood to allow children to use media based on that reason. The highest rated motivation factor to allow infant screen media use for these mothers was the perceived educational benefits of screen time (mean 4.56, SD 1.56), followed by the child asking for screen time for enjoyment (mean 3.76, SD 1.66), and the mother needing to do chores (mean 3.62, SD 1.63). Additional motivation factors that ranked lower included giving an infant a screened device as a reward (mean 3.48, SD 1.74) and to help the infant relax (mean 3.47, SD 1.48).

We also sought to explore whether any differences existed in parental motivation factors based on maternal education level. A dichotomous variable for education level was created with two levels (lesser than a bachelor’s degree and a bachelor’s degree or higher) as an independent variable. There were 62% (53/86) of caregivers in the low education category and 38% (33/86) of caregivers in the high education category. Five dependent variables, consisting of the 5 parental motivation factor subscales, were used in the statistical analysis. There were no outliers in the data based on visual inspection, scores for the factors showed approximately normal distribution based on visual inspection of the Normal QQ Plot, and the assumption of homogeneity of variances was met using Levene test for equality of variances. The significance level for *P* was set at .05, and a series of independent sample *t* tests (2-tailed) were used to assess whether differences existed for any of the parental motivation factors based on maternal education level. Mothers at low education level (lesser than a bachelor’s degree; mean 3.72, SD 1.45) rated the motivation factor *relax* as a rationale for allowing infant screen time higher than mothers at a high education level (mean 3.07, SD 1.46), showing a statistically significant difference of 0.65 (95% CI 0.01-1.29; *t*_84_=2; *P*=.048; Cohen *d*=0.44). A second parental motivation construct, reward, was also rated higher as a reason for allowing screen time by mothers with less education (mean 3.73, SD 1.76) as compared with mothers with high education (mean 3.08, SD 1.66), showing a marginally significant difference of 0.65 (95% CI −0.11 to 1.41; *t*_84_=1.70; *P*=.09; Cohen *d*=0.38; considering a *P* value of .10). None of the other 3 motivation factors differed between the 2 groups by education level, with all *P*>.05.

### RQ 5: Parental Motivation Factors and Infant Screen Time Exposure

The final RQ explored the parental motivation factors for allowing an infant to be exposed to screen time and whether any of the factors predict actual screen time exposure for children. Each of the parental motivation factors was identified as an independent variable for this analysis, with the dependent variable being the average hours of screen time exposure per day (128/178, 71.9%).

Regression analyses were conducted to identify whether any of the parental motivation factors were predictive of screen time exposure during infancy. Results including unstandardized coefficients, SEs, *t* scores, and *P* values are reported in Table 2. None of the motivational factors (ie, educational benefit, chores, reward, relaxation, and asking) predicted daily infant screen time (*F*_5,123_=0.98; *P*=.43; *R^2^*=0.04).

**Table 2:** Linear regressions between average infant screen time per motivational factor.

Parental motivation factor	B (SE)	β	*t* test (*df*)	*P* value
Educational benefit	−0.05 (0.06)	−.10	−0.94 (123)	.35
Ask or enjoyment	0.07 (0.08)	.13	0.90 (123)	.37
Chores	−0.07 (0.07)	−.14	−1.04 (123)	.30
Reward	0.06 (0.05)	.13	1.19 (123)	.24
Relax	0.03 (0.09)	.05	0.32 (123)	.75

## Discussion

### Principal Findings

The overall goal of this study was to determine the degree to which the parent caregivers were aware of the AAP recommendations regarding screen time exposure to infants and toddlers; their adherence to the guidelines; and, if they did not adhere, their reasons for not following the guidelines and any association with infant screen time use. This information can be used by those involved in pediatric, public health, family support, educational, and other settings supporting children and families.

It is important to note that the COVID-19 pandemic could be amplifying or altering the existing discrepancies between the current screen time recommendations and parental awareness, adherence, and motivations for allowing screen time use for infants. Although these data were gathered before the beginning of the pandemic, the Infant Cognitive Development Lab is preparing a manuscript that explores parental motivations during the pandemic period (S Lammers, unpublished data, February 2022).

### Awareness of AAP Screen Time Recommendations

Our initial RQ sought to explore the degree to which parents were aware of the AAP’s guidelines for no sedentary screen time use by infants younger than 24 months (the AAP recommendations during the period the data were collected) [5]. In our results, we found that approximately 62.2% (107/172) of the participants indicated awareness of the guidelines. Previous research has suggested a wide range in parental awareness levels of screen media recommendations, with Funk et al [29] reporting only 34% of parents who were surveyed had an accurate awareness, whereas Adamiak [30] conversely noted that 76% of parents were aware of age-specific media recommendations. Our finding emerged in the upper level of this range but also illustrated the discrepancy in the suggested parental awareness of screen time guidelines, thus establishing an opportunity for more in-depth investigation through an analysis of open-ended responses.

Upon further investigation of participants’ understanding of the AAP guidelines through a qualitative approach, we discovered that only 38.4% (66/172) of mothers were able to accurately state them. However, there were also many mothers who had a general idea that screen time should be limited but were not fully confident of their knowledge (13/172, 7.6%) or aware of the degree to which such restrictions should be applied (17/172, 9.9%). These findings indicate that although most mothers are initially indicating their awareness of the AAP guidelines; a smaller number of them accurately and confidently understand the screen time recommendations for infants younger than 2 years. This pattern suggests the need to reiterate the guidelines in a concise and clear manner with the goal of increasing mothers’ comprehension of the guidelines. Moreover, our results further indicate that maternal awareness of such guidelines is not simply an *either-or* situation, but that there is a range in mothers’ understanding of AAP recommendations.

It is noteworthy that >2 out of 5 mothers in this sample (52/172, 44.2%) were unaware of the AAP’s recommendations, as it suggests there is a continuing lack of awareness about the topic of screen time use during infancy. If we generalize the results regarding maternal awareness from this study to the general adult population of the United States, which has a population of 260 million adults in 2020 [35], we would find that approximately 115 million adults would be unaware of the AAP’s recommendation of no screen time for infants aged <2 years. This finding suggests that current methods of conveying important parenting messages can be improved or expanded. Furthermore, mothers’ understanding of the AAP’s recommendations may also benefit from more elaborate explanations of why screen time should be avoided during infancy rather than simply stating that it should be avoided.

Our study findings also provided insight into how caregivers gain awareness of the AAP guidelines on screen time exposure, with those who were aware of it citing web-based information as a key source (41/172, 23.8%). This is consistent with research suggesting the internet as a common source of parenting information for mothers [25]. This source was closely followed by medical professionals as a primary source of awareness (38/172, 22.1%), a positive finding, as it is consistent with the finding by van der Gugten et al [27] that physician recommendations strongly aid in reducing parental concerns and facilitate the distribution of science-based information to parents in an effective manner [33].

### Adherence to AAP Screen Time Guidelines

This study further explored the level of caregiver adherence to the AAP screen time guidelines for infants, the link between awareness of the guidelines and adherence to them, and whether such adherence influences infant screen time exposure. Although organizations such as the AAP publish such guidelines to encourage best practices in raising children [29], it seems likely that adherence to such recommendations varies widely in actual parental behavior. Findings from the study indicated that approximately half of the parents (86/172, 50%) reported adhering to the recommendations for infants. ANOVA procedures further indicated that adherence to the AAP guidelines resulted in a significant difference in average daily screen time for infants, with those in the *nonadherent* category reporting approximately twice as much infant screen time per day as mothers who followed the guidelines. This finding suggests the potential value of encouraging and facilitating adherence to the AAP recommendations as a mechanism for improving an infant’s well-being. At the same time, as technology is a prominent feature of how contemporary society functions each day, the task of restricting or eliminating access to screens for infants may seem daunting or unrealistic to parents.

In addition, both parent reports of adherence and infant screen time use were related to caregiver awareness of the AAP recommendations. The chi-square analysis indicated that those caregivers who were clearly aware of the AAP recommendations were different in their adherence patterns to screen time recommendations for infants than caregivers who were largely unaware of it. Among caregivers who were aware, approximately 59.8% (64/107) of them adhered to the AAP guidelines with their infant, whereas only approximately 34% (22/65) of mothers who were unaware limited infant screen time exposure. Thus, those who were clearly aware of the guidelines were more likely to restrict screen time for infants. This finding reiterates the need to increase efforts to expand such awareness. Further analysis showed that mothers who were aware of the AAP guidelines allowed significantly less screen time than those who were unaware of the recommendations. However, although awareness seemed to increase compliance with the guidelines, it did not entirely deter parents from allowing some screen time. Our investigation of parental motivations for allowing screen time helped to further explain this finding.

### Parental Motivation Factors and Maternal Education

In circumstances where parents do not adhere to AAP screen time recommendations for infants, we sought to understand the reasons why mothers allow infant screen time exposure and whether maternal education influences these motivations. Previously, research on this topic has suggested a range of parental motivations for allowing screen use by young children [32]. Results of our investigation also revealed that mothers allow screen time for a variety of reasons. Among the 5 parental motivation factors assessed [32], in this study, the highest rated motivation factor was perceived educational benefits for infants (mean 4.56, SD 1.56; on a 7-point scale).

This belief is a moderately troubling misconception. Several studies have demonstrated that infants do not transfer skills they learn on a screened device to the real world, thus furthering the argument for limiting sedentary screen time. In addition, studies with infants show deficits in learning when information is presented in video format rather than from a live individual [1,10]. Hutton et al [3] indicate that instead of providing benefits, infant children exposed to an excess amount of screen time have reduced white matter integrity, which may reduce emerging language skills. However, parents in this instance are giving infants screen time with the belief that they are promoting the well-being of the infant rather than hindering it. Cingel and Krcmar [32] also found that perceived educational benefits was one of the most highly rated motivations for allowing infant screen time in their research, indicating that this belief seems consistent across different groups of parents. However, current research suggests that the usefulness of engaging in screen time activities for infants younger than 2 years might be compared with watching fireworks. Fireworks are flashy and fun to look at, but infants do not learn fundamental skills from watching them. However, unlike fireworks, the use of screen-based media can occupy a significant amount of time in an infant’s life, drawing time away from more worthwhile activities.

In addition, the next two highest parental motivation factors for allowing infant screen time were for infants’ enjoyment of the screened device-based activities (mean 3.76, SD 1.66) and the mothers’ need to do chores around the house (mean 3.62, SD 1.63). These findings were extremely consistent with those of Cingel and Krcmar [32], who also found these 2 motivational factors among the top 3 reasons denoted by parents for allowing infant screen time. When considering such factors, it may be that some parents perceive that they are achieving 2 goals by providing an educational experience for their infants while giving themselves the opportunity to engage in other tasks (eg, cleaning, checking email, and cooking dinner). Similarly, their motivations may encompass multiple reasons at one time, including the factors such as to reward a child or to let a child relax [2,32]. In this study, we added 2 exploratory items that paired with the educational benefits and chores motivational factors, but these additions either did not improve the reliability of the relevant factor or did so only moderately (see *Measures* section).

We also sought to explore whether differences existed among maternal caregivers by education level with regard to how they rated parental motivation factors for allowing infant screen time. Does a mother’s level of education shape her attitudes toward allowing infant screen time when such behavior is discouraged by AAP recommendations? In this study, the 3 motivation factors of educational benefits for infants, the infant’s enjoyment, and doing parental chores were not statistically different by maternal education level. However, mothers who were less educated endorsed infant screen time to reward a child or to help a child relax significantly more than mothers who were more educated (ie, above vs below a bachelor’s degree). This finding may suggest that mothers with a higher education level possess a better understanding of the negative effects associated with screen time exposure during infancy. Therefore, highly educated mothers may be more likely to refrain from using screen time as a tool to calm or reward children, instead using methods that resemble parenting best practices to accomplish these tasks [15,36]. In contrast, the lack of differences in the 3 most highly rated motivational factors seems to indicate that maternal education has a limited influence on mothers’ reasons for allowing infant screen time.

### Parental Motivation Factors and Infant Screen Time

The final RQ explored in this study was whether any of the identified parental motivation factors for allowing infant screen time exposure were predictive of screen time use during infancy. Brown and Smolenaers [2] reported a range of motivations that parents provide for allowing infant screen time, including the child’s enjoyment or as a tool for calming children. However, this study explored 5 specific motivation factors outlined in a measure by Cingel and Krcmar [32]. The regression analysis indicated that none of these parental motivation factors predicted the average daily screen time exposure for infants. Although these factors provide insight into parent motivations in allowing screen time, it seems that other factors such as sibling’s use of a device, number of screens in the household, or other family characteristics will need to be considered to further understand what predicts infant screen time exposure.

### Implications for Education and Policy

The study findings shed light on caregiver awareness of guidelines from the AAP for infant screen time, their adherence to such guidelines, and factors linked with allowing infant screen time exposure. Clearly, the AAP issues such guidelines to educate parents and caregivers, as well as to promote child health and well-being [5]. This study clarifies parental awareness of such information specific to infant screen time, suggesting either a lack of awareness or some level of confusion among many parents regarding the recommended restrictions on screen time for this age group. The fact that many parents knew the guidelines but did not adhere to them is of additional concern.

Among the study findings, it was noted that some parents believe that exposure to screen time before the age of 2 years is actually beneficial to their infant’s well-being and development. Perceived educational benefit was rated by parents as the top motivational factor for allowing infants screen time. Thus, some parents incorrectly conclude that screen time provides opportunities to enhance their infant’s learning, when instead it often replaces the time spent exploring and interacting with their environment—activities that research shows enhance overall development [3-5,12]. Collectively, these findings about the lack of awareness regarding the AAP guidelines, confusion about it, or the belief that infant screen time can be educational suggest that caregivers may benefit from more thorough explanations about why screen time should be avoided during infancy. The implication for those involved in educating parents is that such an effort must go beyond information transmission and instead consider carefully how parents receive information, how to maximize their learning of research-based knowledge, and ways to elevate the impact of this learning in their parenting practices [26,29,33].

In considering policy implications, it seems important to note that the advertisements for many media companies target children in the infancy age range. Parents and caregivers who are uninformed may assume that their children learn from products that promote the use of devices with screens. In some countries, this type of false advertising is banned. Multiple health organizations have made statements that discourage parents from exposing their children younger than 2 years to screen time, including in the United States, Australia, Canada, and France [5,6,9]. Our findings suggest that substantial effort is required to ensure such information is effectively communicated to parents. Policy statements need to be supported by effective communication strategies. A country that has taken extensive policy measures to ensure the reduction of infant media exposure is France. In 2008, the French High Audiovisual Council made the informed decision to ban their television companies from advertising and airing shows aimed at children younger than 3 years [4]. Although research suggests that programs delivered via a device with a screen that target young children do not provide educational value, the marketing of such material is influential, and it may override best practice recommendations by the AAP or similar groups in the minds of parents [4,9]. Alternatively, the marketing of material or products that endorse or encourage infant screen time may be done more effectively than the communication of AAP guidelines on the topic. In either case, this topic represents a growing concern in raising young children and needs to be explored further, as empirical research has demonstrated the adverse effects of screen time exposure on an infant’s development [3,4].

However, it should also be noted that there are contradictory statements regarding screen time use during infancy that perhaps make the decisions around screen time use for infants more confusing and problematic for parents. For example, the Royal College of Pediatrics and Child Health (RCPCH) in the United Kingdom has made policy statements that counter the guidelines set by the AAP and the World Health Organization. The RCPCH [37] believes that the evidence presented on the adverse effects of screen time exposure for infants and young children is often overstated. Instead, the RCPCH directs parents to make their own decisions regarding screen time use based on each individual child, but acknowledge the expert recommendation of avoiding screens 1 hour before bedtime [37]. Understandably, the contradictory statements made by prestigious entities around the world regarding screen time use for infants can make the choice for parents more challenging. Again, this suggests that institutions promoting child well-being should combine policy recommendations with effective communication strategies for reaching parents and reinforcing their key messages.

### Limitations

A few limitations of this study ought to be considered. First, as participants were recruited using a convenience sample, there were limitations in the representativeness of the data. Information was collected in a limited geographic region with a moderately homogeneous population. Therefore, the results may be less generalizable outside the United States or to other regions of the United States. In addition, the sample was limited by restricting eligibility to only female primary caregivers. Additional research with an expanded, more diverse population is advisable to strengthen the understanding of the topic beyond this study.

As with all self-report measures, there is also a possibility of social desirability influencing results, particularly relating to reports of adherence to guidelines and use of screen time. We attempted to reduce this bias by asking questions about the AAP guidelines after we asked participants to estimate screen time, rather than priming them with information about the guidelines. Finally, screen time estimates for this age group may benefit from a more fine-grained analysis, perhaps using increments of 15 minutes instead of the 1-hour range that we used here.

### Conclusions

In summary, this study indicates that mothers of infant children have a mixed awareness of AAP guidelines on screen time. Furthermore, half of the caregivers in this study (86/172, 50%) adhered to the guideline in restricting access to screen time, whereas the other half did not and cited multiple parental motivation factors for allowing infant screen time exposure. Both parental awareness of the AAP guideline and adherence to that guideline were linked with greater likelihood of limiting an infant’s average daily screen time. More highly educated mothers were less likely to endorse certain reasons for allowing infant screen time, such as to help children relax or to reward them, but otherwise, parental motivations for allowing infant screen time did not differ by level of education. Furthermore, parental motivation factors did not predict the average daily screen time exposure of infant children. The findings suggest the importance of extending beyond policy statements to ensure that parents have a clear and informed understanding of recommendations for child well-being that are provided by groups such as the AAP. In doing so, it is hoped that recommendations based on current research can truly be leveraged to enhance parenting best practices and give infant children greater opportunities for enriched learning and positive developmental growth.

## Appendix

Multimedia Appendix 1 Added screening and exploratory questions in the parental motivations scale.

## Abbreviations

AAP: American Academy of Pediatrics
NDSU: North Dakota State University
RCPCH: Royal College of Pediatrics and Child Health
RQ: research question
