# Supplementary material for: Explaining Adherence to American Academy of Pediatrics Screen Time Recommendations With Caregiver Awareness and Parental Motivation Factors: Mixed Methods Study
Source: JMIR Pediatr Parent. 2022 Apr 5;5(2):e29102. doi: 10.2196/29102 (PMC9019621; doi:10.2196/29102)
Supplement: Multimedia Appendix 1 [file pediatrics_v5i2e29102_app1.docx]

APPENDIX A. ADDED SCREENING AND EXPLORATORY QUESTIONS IN THE PARENTAL MOTIVATIONS SCALE

|  | Strongly Disagree | Disagree | Somewhat Disagree | Neither Agree nor Disagree | Somewhat Agree | Agree | Strongly Agree |
| --- | --- | --- | --- | --- | --- | --- | --- |
|  | 1 | 2 | 3 | 4 | 5 | 6 | 7 |
| I let my child use media...  …never (screening variable)  …so I can care for another child (added to chores construct)  …so I can do other things (added to chores construct)  …to benefit their future skill set (added to educational benefit construct) | | | | | | | |
